# A potent bioactive fraction against colon cancer from *Plectranthus vettiveroides*

**DOI:** 10.37349/etat.2023.00131

**Published:** 2023-04-24

**Authors:** Faisal M. Athikkavil, Sreekumar U. Aiswarya, Remya Johny, Meghna Sudhesh, Amrutha A. Nisthul, Ravi S. Lankalapalli, Ruby J. Anto, Smitha V. Bava

**Affiliations:** 1 Molecular Oncology Laboratory, Department of Biotechnology, University of Calicut, Malappuram 673635, India; 2 Division of Cancer Research, Rajiv Gandhi Centre for Biotechnology, Thiruvananthapuram 695014, India; 3 Chemical Sciences and Technology Division, Council for Scientific and Industrial Research (CSIR)-National Institute for Interdisciplinary Science and Technology (CSIR-NIIST), Thiruvananthapuram 695019, India; 4 Academy of Scientific and Innovative Research (AcSIR), Ghaziabad 201002, India; NGO Praeventio, Estonia

**Keywords:** Colorectal cancer, *Plectranthus vettiveroides*, anticancer fraction, apoptosis, protein kinase B

## Abstract

**Aim:**

This study was designed to investigate the anticancer efficacy of the organic leaf extracts of the plant, *Plectranthus vettiveroides* (*P. vettiveroides*), and to analyze the molecular mechanism of the anticancer activity.

**Methods:**

The leaf extracts were prepared by polarity-graded serial extraction of the dried leaf powder. The cytotoxic effect of the extracts was analyzed by the 3-(4, 5-dimethylthiazol-2-yl)-2, 5-diphenyltetrazolium bromide (MTT) assay. The most active ethyl acetate extract was subjected to bioactivity-guided fractionation by column chromatography, which yielded a cytotoxic fraction designated as the *P. vettiveroides* fraction (PVF). The anticancer property of PVF was confirmed further by clonogenic assay. The mechanism of PVF-induced cell death was analyzed by flow cytometry and fluorescence microscopy. Additionally, the effects of PVF on apoptotic and cell survival pathways were analyzed using western immunoblot analysis.

**Results:**

A bioactive fraction PVF, was isolated from the ethyl acetate leaf extract. PVF showed significant anticancer activity against colon cancer cells, whilst normal cells were comparatively less affected. PVF induced strong apoptotic stimuli in colorectal carcinoma cell line HCT116, involving both extrinsic and intrinsic pathways. Investigation into the molecular mechanism of anticancer activity of PVF in HCT116 cells revealed that the fraction activates the pro-apoptotic pathway via tumor suppressor protein 53 (p53) and inhibits the anti-apoptotic pathway by regulating phosphatidylinositol 3-kinase (PI3K) signaling.

**Conclusions:**

The findings of this study demonstrate, with mechanism-based evidence, the chemotherapeutic potential of a bioactive fraction PVF, derived from the leaves of the medicinal plant *P. vettiveroides* against colon cancer.

## Introduction

Primordial communities all over the world have been using the plant realm as an aid for providing a healthier and more sustainable life to humankind [1]. *Plectranthus vettiveroides* (Jacob) N. P. Singh & B. D. Sharma (*P. vettiveroides*), belonging to the family Lamiaceae, is a widely cultivated medicinal plant in southern India. It has been used as a single agent or as an active constituent of several herbal formulations in indigenous medicinal practices of Ayurveda and natural medicine [2–5]. Ethnomedicinal evidence indicates the use of *P. vettiveroides* leaves for digestive ailments such as dyspepsia, dysentery, and ulcers [6]. Various pharmacological properties of the plant have also been reported [7, 8]. The anti-proliferative property of the hydro-alcoholic root extract of *P. vettiveroides* in cancer cells of different tissue origins has been reported [9]. In the present study, a systematic analysis of the anticancer properties of the organic leaf extracts prepared by polarity-graded serial extraction was conducted. Among the three leaf extracts tested (n-hexane, ethyl acetate, and methanol), the ethyl acetate extract showed potent anticancer activity. Cancer cell lines of various tissue origins were screened for their sensitivity towards the ethyl acetate extract, out of which colorectal carcinoma cell line HCT116 (colon) and HeLa (cervical) emerged as the most sensitive. However, considering the use of the plant in traditional medicine as a component of herbal formulations for gut ailments, the efficacy of the extract against colon cancer was investigated.

Colorectal malignancy is the second most lethal and the third most prevalent cancer globally [10]. In 2020, over 1.93 million colorectal cancer cases and more than 0.9 million deaths were reported around the world [11]. In the management of colon cancer, chemotherapy plays a significant role, as it is the choice of treatment for patients with metastasis or unresectable lesions, and is also used in neoadjuvant or adjuvant settings [12]. Current chemotherapy regimen against colorectal cancer includes both single-agent therapies, using 5-fluorouracil, and multiple-agent regimens containing one or several drugs, including oxaliplatin, irinotecan, and capecitabine [13]. Although chemotherapy has improved the overall survival of patients, it is associated with certain limitations, such as systemic toxicity, acquired resistance, and low tumor-specific selectivity [14]. Therefore, targeting molecular pathways involved in cancer cell survival has become an important focal point in the development of novel chemo-therapeutics against colorectal cancers [15]. Various pathways such as β-catenin, tumor suppressor protein 53 (p53), phosphatidylinositol 3-kinase (PI3K)/protein kinase B (PKB, also known as AKT) and mitogen-activated protein kinase (MAPK), have been implicated in the initiation, progression, and migration of colorectal cancer [16, 17]. Intense research is going on to identify novel agents that can interfere with these signaling pathways to induce cell death and inhibit tumor growth.

The present study reports the isolation of a potent anticancer fraction [*P. vettiveroides* fraction (PVF)], from the ethyl acetate extract of *P. vettiveroides*, which is highly efficacious against colon cancer cells having microsatellite instability (MSI) as well as chromosomal instability (CIN) phenotypes. PVF evoked strong pro-apoptotic signals in the colon cancer cell line, HCT116. Further, PVF-induced regulation of the apoptotic as well as cell survival signaling pathways in HCT116 cells was analyzed, and with mechanism-based evidence, its chemotherapeutic potential against colon cancer was demonstrated.

## Materials and methods

### Chemicals and reagents

Cell culture reagents such as Dulbecco's Modified Eagle Medium (DMEM: AT006), Minimum essential medium (MEM: AT154), and 3-(4, 5-dimethylthiazol-2-yl)-2, 5-diphenyltetrazolium bromide (MTT: TC191) were purchased from Hi-Media Laboratories (India). Streptomycin (91014), penicillin (40309), and gentamicin (37636) were purchased from (Sisco Research Laboratories Pvt. Ltd., India). All the chemicals used for phytochemical extraction and column chromatography were obtained from Merck (Germany). Antibodies against poly (ADP-ribose) polymerase (PARP: 9532S), caspase 3 (9662S), caspase 8 (4790S), caspase 9 (9508S), B-cell lymphoma-2 (BCL-2) homology 3-interacting-domain death agonist (BID: 2002T), phospho-AKT (4060T), p53 (9282S), BCL-2 associated X protein (BAX: 2772T), and apoptosis inducing factor (AIF: 5318P) were procured from Cell Signaling Technology (USA). β-actin (A2228), vinculin (V9131) and all other chemicals were purchased from Sigma (USA) if not otherwise indicated.

### Cell lines and culture conditions

The human colorectal carcinoma cell line HCT116, colorectal adenocarcinomas HT-29 and Caco-2, human cervical cancer cell line HeLa, human pancreatic cancer cell line PANC-1, human malignant melanoma cell line A375, mouse fibroblast cell line L929 and human embryonic kidney cell line HEK-293 were procured from the National Centre for Cell Science, Pune, India and maintained in DMEM or MEM containing 10% fetal bovine serum (Gibco 10270) with 55 μg/mL gentamicin, 100 μg/mL streptomycin, 50 U/mL penicillin, and 2 μg/mL amphotericin B. The cells were grown at 37°C in a humidified atmosphere of 5% CO_2_ (BB150, Thermo Scientific) and passaged over two times in a week and confirmed mycoplasma free on regular basis. Exponentially growing cells were taken for all the analysis.

### Plant specimen

The plant specimen, *P. vettiveroides* were collected on 20th June 2019 from the greenhouse (Department of Biotechnology, University of Calicut, India). It was identified and authenticated by Dr. A. K. Pradeep, Curator, Department of Botany, University of Calicut, India. The voucher specimen with an accession number—CALI 7007 was deposited in the herbarium at the same department. The name of the plant has been checked with the “World Flora Online (WFO)” [18].

### Leaf extract preparation and bioactive fraction (PVF) isolation

Organic solvent extracts of the leaf were prepared by successive polarity gradient extraction. Briefly, 25 g of dried leaf powder was treated successively with 500 mL of each solvent (n-hexane, ethyl acetate, and methanol) under room temperature with constant shaking for 24 h. The extracts obtained were dried using a vacuum rotary evaporator. The most cytotoxic extract (ethyl acetate), was subjected to column fractionation by conventional silica gel column chromatography (60–120 mesh, column size 25 cm × 3 cm) using an n-hexane: ethyl acetate solvent system. The crude ethyl acetate extract (500 mg) was loaded on top of the packed silica gel and the column was eluted stepwise with 100 mL of hexane and hexane: ethyl acetate solvent system (100:0), (95:5), (90:10), (85:15), (80:20), (75:25), (70:30), (65:35), (60:40), (55:45), (50:50), (45:55), (40:60), (35:65), (30:70), (25:75), (20:80), (15:85), (10:90), (5:95), (0:100).

The cytotoxic activity of the 82 fractions collected was tested and two cytotoxic fractions (fractions 21 and 22) were identified. As fraction 21 exhibited a better cytotoxic profile, it was considered for further analysis and was designated as PVF.

### MTT assay

The cytotoxic potential of the leaf extracts and PVF was assessed using an MTT assay. Briefly, cells were seeded in 96-well plates (2 × 10^3^ cells/well) other than Caco-2 which was seeded (6 × 10^3^ cells/well) and treated with different concentrations of plant extracts and PVF for 72 h. The drug-containing media was then replaced with fresh media containing 1 mg/mL MTT per well and incubated for 2 h. At the end of incubation, 100 μL of lysis buffer (20% sodium dodecyl sulfate in 50% dimethylformamide) was added to the wells and incubated further for 30 min at 37°C. The optical density was measured at 570 nm using an enzyme-linked immunosorbent assay (ELISA) plate reader (BioTek, Synergy H1). The relative cell viability in percentage was calculated as [A570 (treated cells)/A570 (untreated cells)] × 100. The half-maximal inhibitory concentration (IC_50_) values were extrapolated from polynomial regression analysis of experimental data.

### Clonogenic assay

The proliferative propensity of the HCT116 cells after exposure to PVF was assessed by clonogenic assay. Briefly, 200 cells per well, seeded in a six-well plate were treated with different concentrations of PVF and curcumin (cur: 9 μg/mL) was used as a positive control. After 72 h, the drug-containing medium was replaced with fresh medium, and the plate was incubated for a week. The clones developed were fixed in glutaraldehyde (6%) and stained using crystal violet (0.5%) for 60 min. The number of colonies developed after exposure to PVF was compared to the untreated control.

### Cell morphology analysis

HCT116 and L929 cells were seeded in 35 mm Petri dishes (2 × 10^5^ cells/well), treated with 5 μg/mL and 10 μg/mL PVF for 48 h and the morphological changes in response to PVF were observed under a phase contrast microscope (Carton 100 FS) and imaged.

### Flow cytometry for cell cycle analysis

HCT116 cells treated with different concentrations of PVF (2.5, 5.0, and 10 μg/mL) and cur (9 μg/mL) as positive control, for different time intervals (24 h and 48 h) were harvested, washed with 1X phosphate buffered saline (PBS), and permeabilized with 70% ice-cold ethanol for 30 min. The permeabilized cells were then treated with 100 mg/mL RNAase A and 50 mg/mL propidium iodide (PI) and subjected to flow cytometric analysis [fluorescence-activated cell sorting (FACS) Aria™, BD Bioscience].

### Annexin V-PI staining

Apoptotic cells in response to PVF treatment were visualized with the help of fluorescent microscopy by Annexin V apoptosis detection kit following the manufacturer's instructions (BD Pharmingen 556547, BD Biosciences, USA). Briefly, the HCT116 cells were seeded in a 96-well plate and treated with different concentrations of PVF (2.5 μg/mL and 5.0 μg/mL), and cur (9 μg/mL) as positive control for 24 h. The cells were washed with 1X PBS and 1X assay buffer and incubated for 15 min with 5 μL of Annexin V fluorescein isothiocyanate (FITC) and 10 μL of PI. At the end of incubation, following a PBS wash, fluorescent images were captured using an inverted fluorescence microscope (Nikon TE-Eclipse 300).

### Fluorescent microscopy to detect reactive oxygen species

Reactive oxygen species (ROS) levels within the HCT116 cells in response to PVF treatment were observed by 2′, 7′-dichlorodihydrofluorescein diacetate (H2DCF-DA) staining as per the manufacturer's protocol. Briefly, the cells were seeded in 60 mm dishes and treated with different concentrations of PVF for 6 h. Post-treatment, the cells were harvested and incubated in dichlorodihydrofluorescein diacetate (DCFDA) containing assay buffer for 30 min. The fluorescence developed was imaged and quantified using an inverted fluorescence microscope (Nikon TE-Eclipse 300). Tert-butyl hydroperoxide (TBHP) 50 μmol/L was used as a positive control.

### Immunoblot analysis

Briefly, 5 × 10^5^ cells were seeded and treated with different concentrations of PVF (2.5, 5.0, and 7.5 μg/mL), and cur (9 μg/mL) for 24 h. The whole-cell lysates prepared (60–80 μg) were electrophoresed by sodium dodecyl sulfate-polyacrylamide gel electrophoresis (SDS-PAGE) and blotted onto polyvinylidene difluoride (PVDF: Merck IPVH00010) membranes. Each membrane was blocked with 5% skimmed milk for 60 min and probed with labeled primary antibodies against caspase 8, caspase 9, caspase 3, PARP, BID, AIF, p53, BAX, and phospho-AKT. The bands were visualized by staining with 3, 3′-diaminobenzidine or enhanced chemiluminescence reagent [19].

### Statistical analysis

The data analysis of flow cytometry was performed through BD FACS Diva software version 5.0.2. All the other statistical analysis was carried out using GraphPad Prism (version 8.0, San Diego, CA, USA) and the quantification of the immunoblot data was performed using ImageJ software (version 1.53 K). Statistical significance was defined as *P* < 0.05. The error bars represent standard deviation (SD), taken from three independent experiments.

## Results

### PVF, a bioactive fraction isolated from *P. vettiveroides* leaves is highly efficacious against colorectal cancer cells

**Figure 1.**
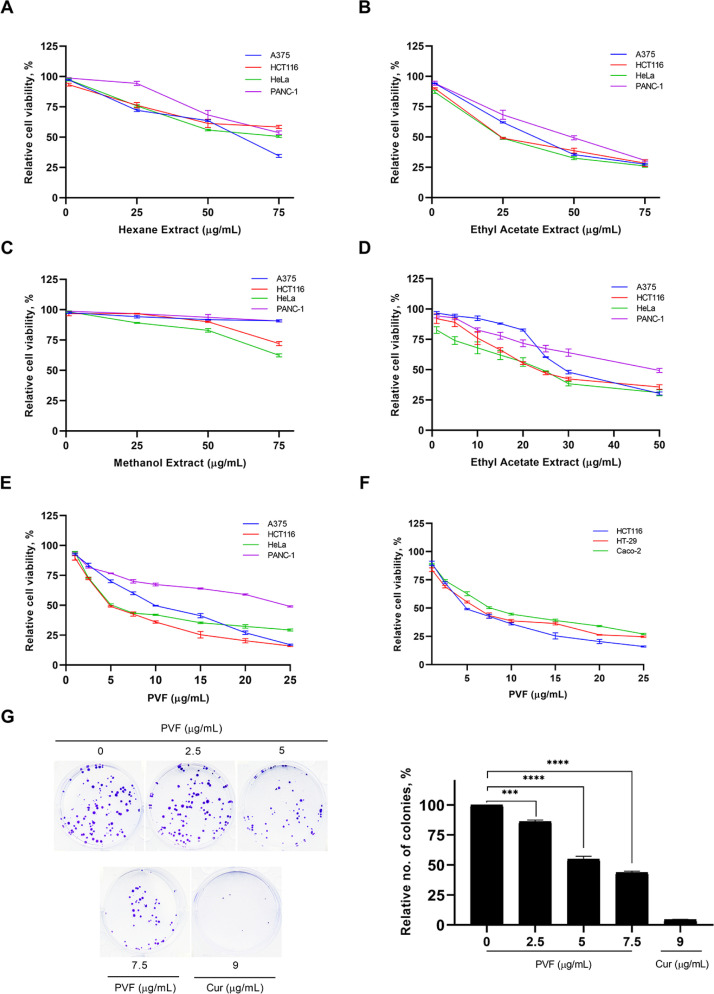
PVF, a bioactive fraction from *P. vettiveroides* is highly efficacious against colon cancer cell lines. (A–C) Screening for the cytotoxic potential of n-hexane, ethyl acetate, and methanol leaf extracts in cancer cell lines of various tissue origins. Various concentrations of leaf extracts (1, 25, 50 and 75 μg/mL) were used for the experiment; (D) determination of IC_50_ value of ethyl acetate leaf extract in cancer cell lines of various tissue origins. Various concentrations of ethyl acetate leaf extracts (1, 5, 10, 15, 20, 25, 30 and 50 μg/mL) were used for the experiment; (E) determination of IC_50_ value of the bioactive fraction obtained from the ethyl acetate leaf extract (PVF) in cancer cell lines of various tissue origins; (F) colon cancer cell lines. Various concentrations of PVF (1, 2.5, 5, 7.5, 10, 15, 20 and 25 μg/mL) were used for the experiments; (G) PVF inhibits the proliferative potential of HCT116 cells as assessed by clonogenic assay. Cur 9 μg/mL was used as a positive control. Data are representative of three independent experiments [mean ± standard error of mean (SEM)] and *P*-values are calculated using one way ANOVA. ^****^ *P* ≤ 0.0001 and ^***^ *P* ≤ 0.001; no.: relative number of colonies

To explore the anticancer potential of the plant *P. vettiveroides*, we conducted serial exhaustive extraction of the shade-dried leaf powder of the plant, using three organic solvents based on increasing polarity (n-hexane, ethyl acetate, and methanol). All three extracts obtained were tested for the cytotoxic potential in cancer cell lines of various tissue origins using an MTT assay (Figure 1A–C). The results revealed ethyl acetate extract as the most potent, exhibiting notable cytotoxicity with an IC_50_ value of about 25 μg/mL specifically in the carcinoma cell lines, HCT116 (colon) and HeLa (cervical) (Figure 1D). To isolate the anticancer principle(s) from the ethyl acetate extract, we performed silica gel column chromatography, tested the isolated fractions for cytotoxic activity, and identified a potent cytotoxic fraction, which was designated as “PVF” (Figure S1 and Figure S2, systematic PVF isolation has been detailed in the methodology). Further, we investigated the cytotoxic efficacy of the bioactive fraction, PVF against various human cancer cell lines viz. A375 (melanoma), HCT116 (colon), HeLa (cervical), and PANC-1 (pancreas) using MTT assay. Interestingly, in line with the cytotoxicity profile obtained for ethyl acetate extract, PVF bioactive fraction induced significant cytotoxicity in the colorectal cancer cell line, HCT116, and the cervical cancer cell line, HeLa (IC_50_—5 μg/mL) (Figure 1E). As *P. vettiveroides* is used in traditional medicines for gut-related ailments, we continued our studies using colon cancer cell lines. Firstly, we confirmed the increased sensitivity of colon cancer cells towards PVF by screening different colorectal cancer cell lines of two molecular subgroups, viz. cell lines with CIN phenotype (HT-29 and Caco-2) and MSI phenotype (HCT116). Interestingly, all the colorectal cancer cell lines studied exhibited considerable sensitivity toward PVF. The cell line, HCT116 was repeatedly observed as the most sensitive to PVF and was considered for further studies (Figure 1F). We also confirmed the potential of PVF to inhibit the proliferative propensity of HCT116 cells using the clonogenic assay, keeping cur (9 μg/mL) as a positive control. As expected, the number and size of colonies produced were significantly reduced in the PVF-treated plates, as compared to that of the untreated control, confirming the efficacy of PVF against colon cancer (Figure 1G).

**Figure 2.**
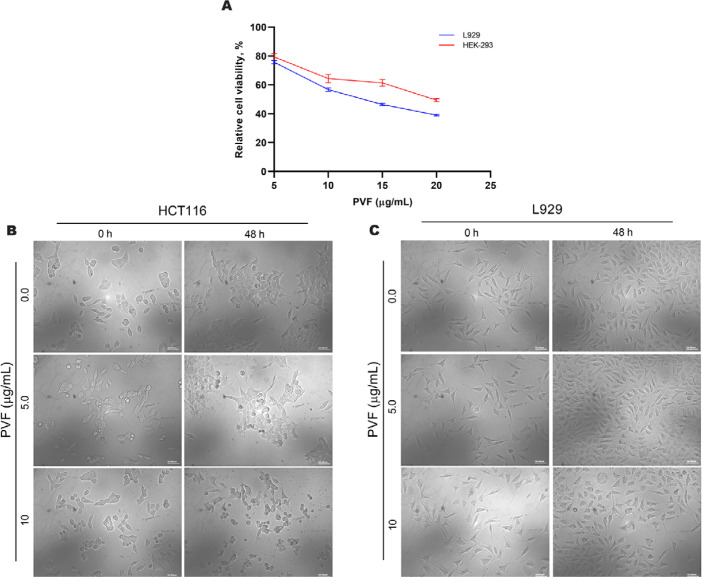
PVF is less toxic to normal cells. (A) Cytotoxicity assessment of PVF in normal immortalized L929 mouse fibroblast cells and HEK-293 human embryonic kidney cells; (B) morphological changes in response to PVF in HCT116 cells; (C) L929 cells (20× magnification and scale bar = 50 μm). Data are representative of three independent experiments and the (mean ± SEM) were calculated

We further tested the biological safety of PVF, by studying its effect on the normal immortalized cell lines viz. mouse fibroblast L929 and human embryonic kidney HEK-293 cells, using MTT assay. Interestingly, the result revealed that the IC_50_ concentration of PVF in these cells is three times and four times higher than that in HCT116 cells for L929 and HEK-293 respectively (Figure 2A). Morphological examination of HCT116 and L929 cells treated with 5 μg/mL and 10 μg/mL of PVF for 48 h, clearly showed that L929 normal cells are not affected even after prolonged treatment with a concentration i.e. twice the IC_50_ in HCT116 cells, confirming the non-toxic nature of the fraction on L929 cells (Figure 2B and 2C). These data demonstrate that the potent bioactive fraction PVF acquired from *P. vettiveroides* is highly efficacious against colorectal cancer cells, whilst being less toxic to normal cells.

### PVF induces an apoptotic mode of cell death in colorectal cancer HCT116 cells

**Figure 3.**
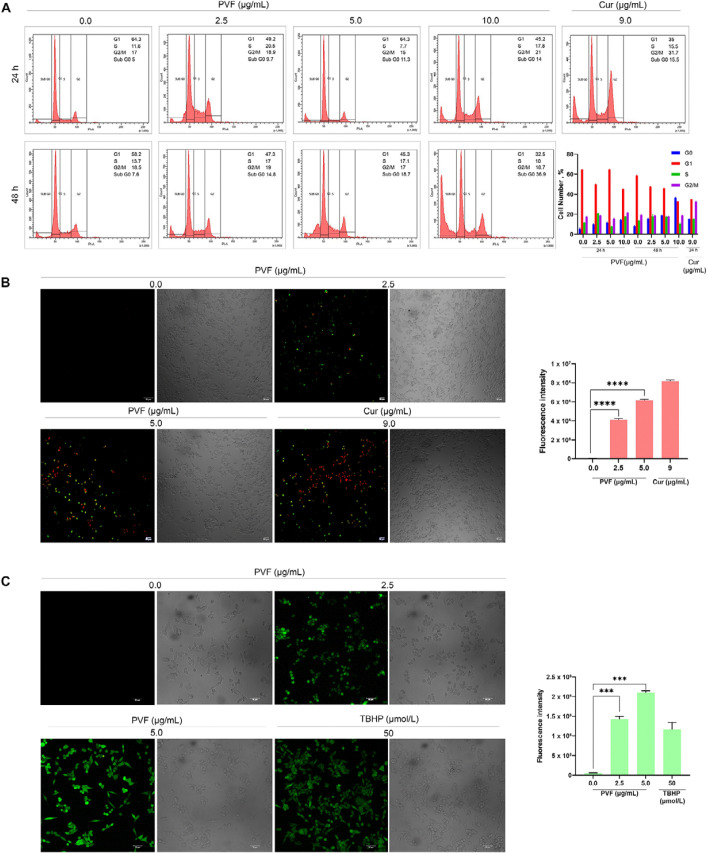
Analysis of the PVF-induced cytotoxic mechanism in HCT116 cells. (A) PVF does not affect any phases of the cell cycle in HCT116 cells as demonstrated by flow cytometry; (B) the extent of apoptosis induced by PVF in HCT116 cells by Annexin V/PI staining (10× magnification and scale bar = 50 μm). Cur 9 μg/mL was used as a positive control; (C) ROS production in response to PVF treatment in HCT116 cells as detected by fluorescence microscopy (20× magnification and scale bar = 50 μm). TBHP 50 μmol/L was used as a positive control. Data are representative of three independent experiments (mean ± SEM) and *P*-values are calculated using one-way ANOVA. ^****^ *P* ≤ 0.0001, ^***^ *P* ≤ 0.001, and ^**^ *P* ≤ 0.01

We further investigated the mode of cell death induced by PVF in HCT116 cells. Firstly, we analyzed whether PVF induces cell cycle arrest, by flow cytometry analysis. However, PVF did not influence any stages of the cell cycle, even after prolonged treatment for 48 h at IC_50_ 5 μg/mL and 10 μg/mL (twice the IC_50_ concentration) (Figure 3A). Interestingly, there was a considerable rise in the number of cells in the sub-G0 phase, which is an indication of apoptosis. To assess apoptotic induction by PVF in HCT116 cells, we performed Annexin V FITC/PI staining. Indeed, upon treatment with PVF, a substantial increase in the number of apoptotic cells stained with FITC/PI+ was noticed compared to that of the untreated control. Cur (9 μg/mL) was used as the positive control (Figure 3B). Many chemotherapeutic drugs currently used in the clinic, invoke apoptosis in cancer cells via ROS-mediated deoxyribo nucleic acid (DNA) damage and p53 activation [20]. Therefore, we tested whether PVF induces ROS generation in HCT116, for which we used ROS-sensitive H2DCF-DA fluorescence assay. Interestingly, PVF treatment in HCT116 cells triggered ROS production as evidenced by the oxidation of H2DCF-DA to dichlorofluorescein (DCF) which led to the generation of green fluorescence, the intensity of which was measured by confocal microscopy (Figure 3C). Together, these results indicated that the cytotoxic mechanism exhibited by PVF involves ROS production, subsequent DNA damage, and apoptosis.

### PVF induces caspase-dependent apoptosis involving intrinsic and extrinsic pathways in HCT116 cells

**Figure 4.**
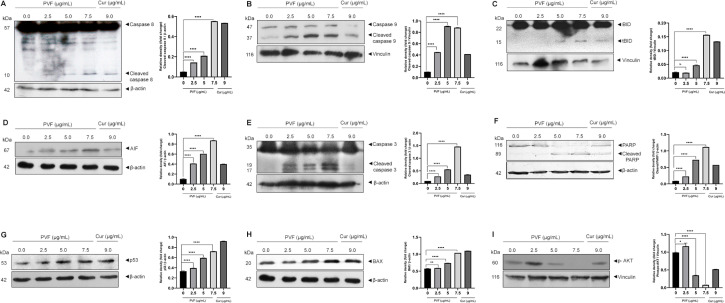
PVF induces apoptosis involving intrinsic and extrinsic pathways in HCT116 cells. (A–F) PVF potentiates the activation of caspases 8, 9, cleavage of BID, AIF release, cleavage of caspase 3 and PARP in HCT116 cells as analyzed by immunoblotting; (G, H) PVF induces p53 and BAX activation in HCT116 cells; (I) PVF inhibits phospho-AKT (p-AKT) in HCT116 cells. Cur 9 μg/mL was used as a positive control. Data are representative of three independent experiments (mean ± SEM) and *P*-values are calculated using one-way ANOVA. ^****^ *P* ≤ 0.0001, ^**^ *P* ≤ 0.01, ^*^ *P* ≤ 0.1 and ns: *P* ≥ 0.05

To further confirm apoptosis and delineate apoptotic pathways in response to PVF in HCT116 cells, we examined the activation of caspases by western immunoblot analysis. Apoptosis in cells is mediated by caspases belonging to the cysteine-aspartic proteases family, classified as initiators and executioners based on their function. The initiator caspases, caspase 8 and caspase 9, activated in response to extrinsic and intrinsic apoptotic stimuli respectively, lead to subsequent activation of executioner caspases, resulting in the cleavage of the cellular death substrates, causing the cells to undergo apoptosis. Upon treatment with PVF in HCT116 cells, we observed both the initiator caspases 8 as well as 9 activations in a dose-dependent manner (Figure 4A and 4B).

Additionally, we looked for BID activation, a particular proximal substrate of caspase 8 in the extrinsic apoptotic signaling cascade, and a regulator of caspase 8-induced mitochondrial stimulation. Remarkably, we noticed activation of BID upon PVF treatment (Figure 4C), demonstrating amplification of apoptotic signals generated at the death receptor through the mitochondria-mediated intrinsic pathway of apoptosis. Indeed, we observed increased expression of AIF in response to PVF in a dose-dependent manner, demonstrating mitochondrial membrane damage and release of AIF (Figure 4D). Furthermore, PVF induced cleavage of the executioner caspase, caspase 3, and its classical substrate, PARP (Figure 4E and 4F). Conclusively, these data demonstrate that the bioactive fraction (PVF) from *P. vettiveroides*, induces caspase-dependent apoptosis involving both the extrinsic and intrinsic pathways, in HCT116 cells.

Our next attempt was to investigate the molecular mechanism of the anticancer activity of PVF in HCT116 colon cancer cells. Colon cancer cells of MSI phenotype are reported to show resistance to DNA-damaging agents [21]. Proteins in the DNA mismatch repair (MMR) system are believed to be essential for DNA damage-induced p53 activation and subsequent cell cycle arrest or apoptosis [22]. As HCT116 is an MSI, with wild-type p53, we looked for p53 activation in response to PVF by western immunoblot analysis. Curiously, we observed increased p53 levels in response to PVF in a dose-dependent manner in HCT116, despite being MMR mutant (Figure 4G). We further looked for the transcriptional activity of p53 by analyzing the expression levels of the pro-apoptotic protein BAX, the transcription of which is regulated by p53 [23]. The result of immunoblot analysis showed increased expression levels of BAX protein in response to PVF in a dose-dependent manner, confirming the increased transcriptional activity of p53 (Figure 4H). The data demonstrate DNA damage-induced p53 signaling in response to PVF-induced ROS generation, in HCT116 cells, despite being MMR mutant. Thus, it can be concluded that strong pro-apoptotic signals initiated extrinsically as well as intrinsically in response to PVF in HCT116 drive the cells toward apoptosis. Further, we investigated whether PVF is regulating the anti-apoptotic PI3K signaling in HCT116 by immunoblot analysis of p-AKT. p-AKT inhibits apoptosis by phosphorylation inactivation of pro-apoptotic BAX and BCL-2 associated agonist of cell death (BAD) [24]. It also inactivates the forkhead box O (FOXO) transcription factor, thus down-regulating the expression of BCL-2 interacting mediator of cell death (BIM) and Fas cell surface death receptor (FAS) ligands [25]. Interestingly, we found a significantly high level of p-AKT in HCT116 cells, indicating strong anti-apoptotic signaling, which could make the cells resistant to chemotherapy [26]. However, PVF treatment almost completely ablated the p-AKT band, in HCT116 cells, as assessed by immunoblot analysis (Figure 4I). To conclude, the data clearly shows the ability of PVF in promoting apoptosis in HCT116, by inducing strong proapoptotic signals and inhibiting anti-apoptotic signaling.

## Discussion

Insights from traditional medicine have made significant contributions to modern medicine, especially in the field of cancer chemotherapy [27]. The present study has analyzed the anticancer property of the plant *P. vettiveroides*, which is recommended by the Indian Ayurvedic medicine system for the treatment of various gut-related ailments [2, 6]. Recently, acetone extracts prepared from the *Plectranthus* genus were analyzed for various bioactive properties and anticancer activity of *Plectranthus hadiensis* and *Plectranthus ciliates* have been reported [28]. However, except for a report indicating the anti-proliferative property of the hydro-alcoholic root extract of *P. vettiveroides* in cancer cells of different tissue origins, there are no studies on the anticancer potential of the plant [9]. Hence, the present work has systematically analyzed the anticancer property of the leaf extracts prepared by polarity-graded serial extraction of the shade-dried, powdered leaves of the plant, and the ethyl acetate extract was identified as the most potent. Further, we isolated a cytotoxic fraction, PVF from ethyl acetate extract, which showed significant anticancer activity against colon cancer cells. Colorectal cancer is the third-most common cancer worldwide with about 0.935 million deaths reported in 2020 [11]. Chemotherapy regimens involving general cytotoxic drugs like 5-fluorouracil, oxaliplatin, and irinotecan are of limited success due to acquired resistance, lack of tumor-specific selectivity, and associated toxicity. Moreover, targeted therapy using specific small molecule inhibitors against molecular targets has its limitations, owing to complex downstream signaling and difficulties in completely inhibiting specific biological interactions [29, 30]. Furthermore, all pathways regulating the signaling events in colorectal cancer cannot be successfully impeded by specific target agents [31]. Rather than targeting a specific molecule, our approach is to explore natural sources to identify active fractions or compounds that would target cancer cells of a specific type [19]. These bioactive molecules or fractions exert an anticancer effect by modulating multiple cellular signaling pathways, inhibiting cell proliferation and/or activating cell death signals, leading to apoptosis of cancer cells [32].

PVF, the bioactive fraction isolated from *P. vettiveroides*, induced apoptotic mode of cell death in the colon cancer cell line, HCT116. We observed activation of both the initiator caspases, caspase 8 and 9 as well as the executioner caspase 3, which lead to cleavage of PARP, a major hallmark of apoptosis, indicating the involvement of extrinsic as well as intrinsic pathways. PVF-induced cleavage and activation of BID, in HCT116 cells, demonstrated the amplification of signals from the death receptor of extrinsic pathway, via truncated BID (tBID) induced activation of BAX and BCL-2 antagonist/killer (BAK), which stimulates mitochondria invoking intrinsic pathway of apoptosis. This observation was substantiated by the release of AIF from mitochondria, in response to PVF in HCT116 cells. Many cancer therapeutics generate ROS, which induces DNA damage and subsequent apoptotic cell death in cancer cells [20]. Incontrovertibly, the greater but non-lethal concentration of ROS in cancer cells compared to normal cells promotes cancer cell survival and tumor growth [33]. In this context, chemotherapeutic drugs that generates even more ROS can make the situation critical, thus selectively leading cancer cell to death [20]. Indeed, PVF triggered ROS production in HCT116 cells. Moreover, at the IC_50_ concentration of PVF in HCT116 (5 μg/mL), PVF was found to be non-toxic to normal immortalized cell lines, viz. L929 and HEK-293. Interestingly, PVF induced 50% cell death in L929 and HEK-293, only three times and four times its IC_50_ concentration in HCT116, respectively.

DNA MMR system proteins are believed to be involved in DNA damage-induced cell cycle arrest/apoptosis through the activation of p53 [22]. Cancer cells deficient in MMR are reported to show resistance to apoptosis triggered by DNA-damaging agents/drugs [21]. However, our study reveals that PVF induces apoptosis in HCT116, even though the cell line exhibits MSI phenotype due to an impaired MMR function [34]. As p53 is involved in MMR-mediated DNA damage signaling, the ability of PVF to induce an extrinsic pathway of apoptosis might explain the efficacy of PVF against HCT116. This could also be the reason for the sensitivity of the CIN colon cancer cells, HT-29 and Caco-2 with mutant p53 phenotype, towards PVF. The accumulation of PVF-induced DNA damage in the absence of p53 signaling in the context of CIN and MSI might exacerbate the situation in cells, tipping the balance in favor of apoptotic cell death initiated at the cell surface and amplified through mitochondria. However, in HCT116 cells, we observed significant up-regulation of the p53 protein and its downstream pro-apoptotic protein, BAX, indicating the stabilization and accumulation as well as transcriptional activity of p53 [23]. This result indicates activation of p53 in response to DNA damage in HCT116 cells, which possess wild-type p53, despite having MSI phenotype and impaired MMR function. In addition, PVF has been found to down-regulate the anti-apoptotic signaling via PI3K by inhibiting the AKT phosphorylation in these cells. One of the prominent reasons for resistance to chemotherapeutic agents currently used in the clinic against colon cancer is decreased apoptotic responsiveness by activation of anti-apoptotic pathways, the most important being the PI3K signaling [26]. AKT exerts its anti-apoptotic effect by the phosphorylation inactivation of the pro-apoptotic proteins, BAX and BAD, which mediates mitochondria-mediated apoptosis [24]. AKT also blocks FOXO-mediated transcription of pro-apoptotic genes BIM and FAS ligand. BIM binds to and inhibits the anti-apoptotic proteins of the BCL-2 family, while FAS ligand mediates the extrinsic pathway of apoptosis [25]. Thus, PVF shows promise in the treatment of AKT-positive colon cancers.

To summarize, our study has identified a potent anticancer fraction (PVF), from the plant *P. vettiveroides*, which is highly efficacious against colon cancer cells. The fraction induced strong apoptotic signals in the context of the mutator phenotype of colon cancer cells, leading the cells to apoptosis involving both extrinsic and intrinsic pathways. Molecular investigations into the anticancer mechanism of PVF, in the colon cancer cell line HCT116, revealed the activation of apoptotic signaling via tBID and p53 as well as the inhibition of the anti-apoptotic PI3K signaling via AKT. To conclude, we demonstrate, with mechanism-based evidence, the chemotherapeutic potential of PVF, a bioactive fraction derived from the leaves of the medicinal plant, *P. vettiveroides* against colon cancer.

## Abbreviations

AIF: apoptosis inducing factor
BAX: B-cell lymphoma-2 associated X protein
BCL-2: B-cell lymphoma-2
BID: B-cell lymphoma-2 homology 3-interacting-domain death agonist
BIM: B-cell lymphoma-2 interacting mediator of cell death
CIN: chromosomal instability
Cur: curcumin
DNA: deoxyribo nucleic acid
FAS: Fas cell surface death receptor
FITC: fluorescein isothiocyanate
H2DCF-DA: 2′, 7′-dichlorodihydrofluorescein diacetate
IC_50_: half-maximal inhibitory concentration
MMR: mismatch repair
MSI: microsatellite instability
MTT: 3-(4, 5-dimethylthiazol-2-yl)-2, 5-diphenyltetrazolium bromide
*P. Vettiveroides*: *Plectranthus vettiveroides*
p53: tumor suppressor protein 53
PARP: poly (ADP-ribose) polymerase
PBS: phosphate buffered saline
PI: propidium iodide
PI3K: phosphatidylinositol 3-kinase
PKB: protein kinase B
PVF: *Plectranthus vettiveroides* fraction
ROS: reactive oxygen species
SEM: standard error of mean

## Supplementary materials

The supplementary material for this article is available at: https://www.explorationpub.com/uploads/Article/file/1002131_sup_1.pdf.

## Declarations

### Availability of data and materials

All relevant data are contained within the manuscript and supplementary data.

### Copyright

© The Author(s) 2023.
