# Supplementary material for: A potent bioactive fraction against colon cancer from Plectranthus vettiveroides
Source: Explor Target Antitumor Ther. 2023 Apr 24;4(2):227–39. doi: 10.37349/etat.2023.00131 (PMC10185442; doi:10.37349/etat.2023.00131)
Supplement: Supplementary file 1 [file etat-04-1002131_sup_1.pdf]

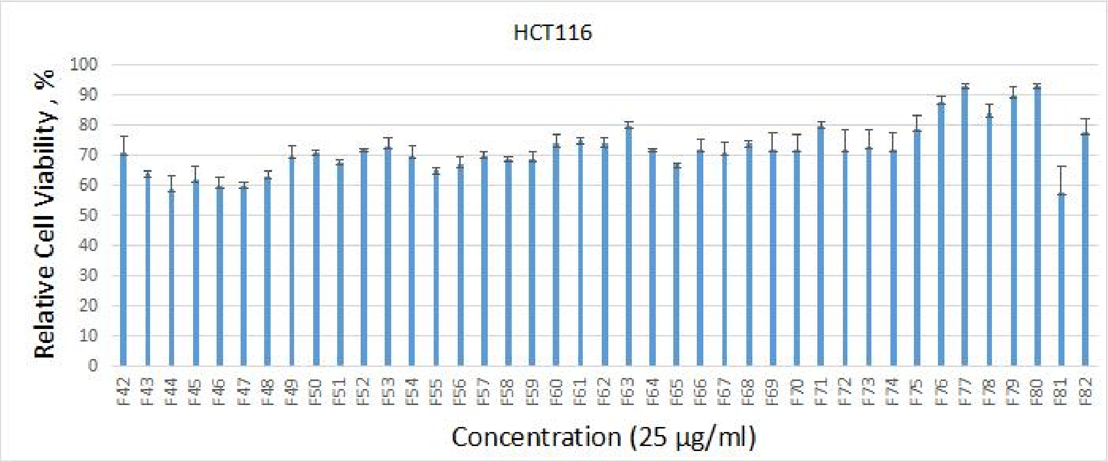

Figure S1. Evaluation of the cytotoxicity of fractions1-41 using MTT assay

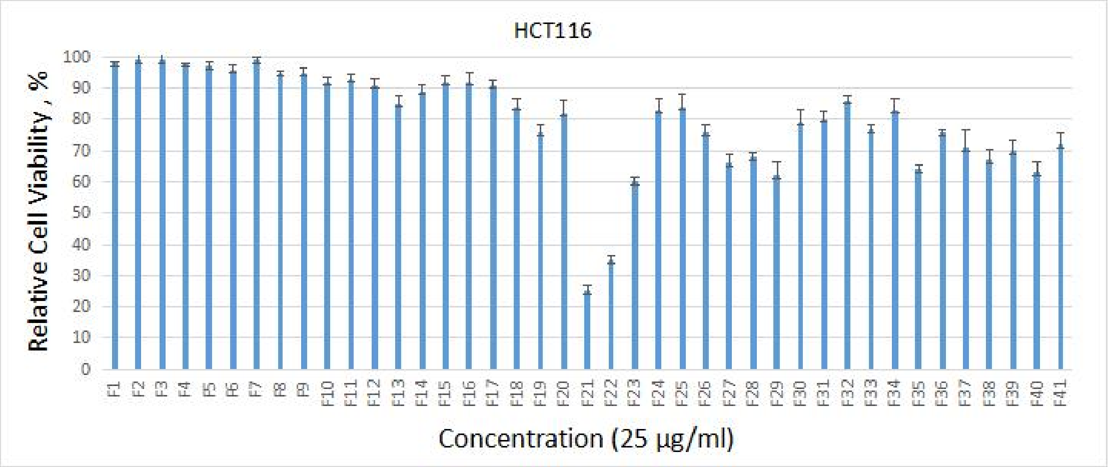

Figure S2. Evaluation of the cytotoxicity of fractions 42-82 using MTT assay
